# Fetal Echocardiography After a Normal Anatomy Assessment: A Retrospective Cohort of In Vitro Fertilization Pregnancies

**DOI:** 10.1111/echo.70655

**Published:** 2026-10-24

**Authors:** Brianna Dickson, Hannah Maher, Savannah Maynard, Rachel Scalf, Madison Sundlof, Katelyn M. Tessier, Daniel Peck, Matthew Ambrose, Cresta W. Jones

**Affiliations:** ^1^ Department of Obstetrics, Gynecology, and Women's Health University of Minnesota Minneapolis Minnesota USA; ^2^ University of Minnesota Medical School Minneapolis Minnesota USA; ^3^ Biostatistics Core, Masonic Cancer Center University of Minnesota Minneapolis Minnesota USA; ^4^ Division of Pediatric Cardiology, Department of Pediatrics University of Minnesota Minneapolis Minnesota USA

**Keywords:** congenital heart disease, fetal echocardiography, in vitro fertilization, obstetric ultrasound, singleton pregnancy, ventricular septal defect

## Abstract

**Purpose:**

To investigate the detection rate of congenital heart disease (CHD) on fetal echocardiogram after a normal and complete detailed fetal ultrasound among pregnancies conceived by in vitro fertilization (IVF).

**Methods:**

This retrospective cohort study examined singleton IVF pregnancies that underwent a detailed fetal anatomic survey without any identified abnormalities, followed by fetal echocardiogram. The primary outcome was the rate of CHD diagnosis after normal and complete anatomic ultrasound. Patients with multifetal gestation, pre‐gestational diabetes, a known family history of CHD, fetal abnormalities on anatomy ultrasound, or patients who had an incomplete initial anatomy ultrasound were excluded. Prenatal CHD was confirmed with neonatal imaging and infants were followed after birth.

**Results:**

300 pregnancies were included, and of these 266 had a normal and complete anatomy ultrasound. 1.5% (*n* = 4) of pregnancies had minor CHD identified on fetal echocardiography which was not identified on the anatomy ultrasound. None of the infants required cardiac‐related interventions in the first year of life.

**Conclusion:**

No major CHD was identified at fetal echocardiography after a normal and complete anatomic survey. Our results support the American Institute of Ultrasound in Medicine guidelines that state a dedicated fetal echocardiogram may be considered, but is not recommended, for singleton IVF pregnancies.

## Graphical abstract

In 266 singleton in vitro fertilization (IVF) pregnancies with no additional risk factors for congenital heart disease (CHD), only 4 cases of minor CHD were detected by fetal echocardiography after a normal anatomy ultrasound. Fetal echocardiography may not be necessary for all IVF pregnancies that are otherwise low risk for CHD.

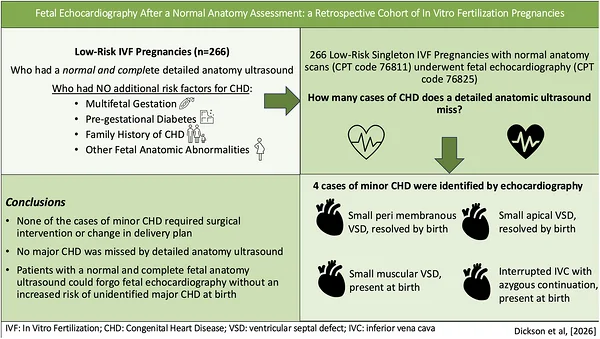

**Abbreviations**
IVF: in vitro fertilization
CHD: congenital heart disease
AIUM: American Institute of Ultrasound in Medicine
AHA: American Heart Association
SMFM: Society for Maternal‐Fetal Medicine
ASE: American Society of Echocardiography
CPT: current procedural terminology
MFM: maternal‐fetal medicine
VSD: ventricular septal defect

## 1 Introduction

Pregnancies conceived by in vitro fertilization (IVF) have historically been shown to be at increased risk for congenital heart disease (CHD) [1]. In light of this, prior society guidelines from the American Institute for Ultrasound in Medicine (AIUM), American Heart Association (AHA) and the Society for Maternal‐Fetal Medicine (SMFM) recommended routine fetal echocardiogram in addition to a detailed fetal anatomic ultrasound examination in this patient population [2, 3, 4]. However, recent studies have questioned this clinical practice recommendation, instead proposing that the increased CHD risk among pregnancies conceived by IVF may be attributed to higher rates of multifetal gestation and the association between twinning and CHD [5, 6, 7, 8, 9]. A large‐scale cohort study found no clinically significant difference in CHD risk between IVF pregnancies without known risk factors and the general population, suggesting a potential role for risk‐based screening [5].

The cost effectiveness of routine fetal echocardiograms in IVF pregnancies has also been questioned, with research suggesting selective fetal echocardiography only in the setting of abnormal findings on routine detailed ultrasound examination [10]. As a result of recently published data, the American Society of Echocardiography (ASE), AIUM, AHA and SMFM updated their guidelines to state that fetal echocardiography “may be considered” in IVF pregnancies. The guidelines further qualify that the decision to perform fetal echocardiogram in the absence of maternal or fetal risk factors and normal detailed ultrasound examination should be based on the specificity and sensitivity of CHD detection practices in the community [11]. In rural, under‐resourced communities, prioritizing higher risk patients for echocardiograms may help to improve patient‐centered care.

An additional practical consideration for patients is their ability to obtain a complete fetal assessment with one anatomy ultrasound visit rather than having to also undergo an additional visit for a fetal echocardiogram, possibly with a referral to another medical practice. Even if a patient is receiving obstetric care with an obstetrician who can perform the more detailed anatomy ultrasound (Current Procedural Terminology [CPT] code 76811), they may then need to be referred to another team for the echocardiogram, as these are usually performed by pediatric cardiologists or maternal‐fetal medicine (MFM) specialists. Multiple visits for fetal evaluation requires more time at clinical appointments and can be anxiety‐inducing to patients, even when they are referred only for further visualization of the fetal heart [12]. Identifying patients who might be able to forgo a fetal echocardiogram could help limit multiple imaging visits, the associated medical costs, and patient inconvenience.

This study aims to investigate the utility of fetal echocardiography in the setting of singleton IVF pregnancies after a normal and complete detailed ultrasound examination has already been performed. We hypothesized that a fetal echocardiogram performed after a normal and complete detailed ultrasound examination, including a normal and complete cardiac assessment, would not identify any additional major cardiac anomalies among IVF pregnancies without additional risk factors for CHD.

## 2 Materials & Methods

This is a retrospective cohort study using an electronic obstetric ultrasound database from a single academic health system. Singleton IVF pregnancies from January 1, 2017, to December 31, 2021, who underwent both an initial completed and normal fetal anatomic survey and subsequently underwent a fetal echocardiogram were eligible for inclusion. Multifetal gestation, pre‐gestational diabetes, abnormal genetic screening (cell free fetal DNA screen, quad marker screen, first trimester screen) or genetic testing (chorionic villus sampling, amniocentesis), or a reported family history of CHD were excluded in order to target the low CHD risk population. Following the application of these criteria, patients with fetal abnormalities, defined as any fetal anatomic structural difference, identified on detailed ultrasound examination were also excluded (Figure 1). Potential participants who did not consent to the use of their data for research were excluded. This study was approved by a local Institutional Review Board. The Strengthening the Reporting of Observational Studies in Epidemiology (STROBE) checklist was utilized in the creation of this study report [13].

**FIGURE 1:**
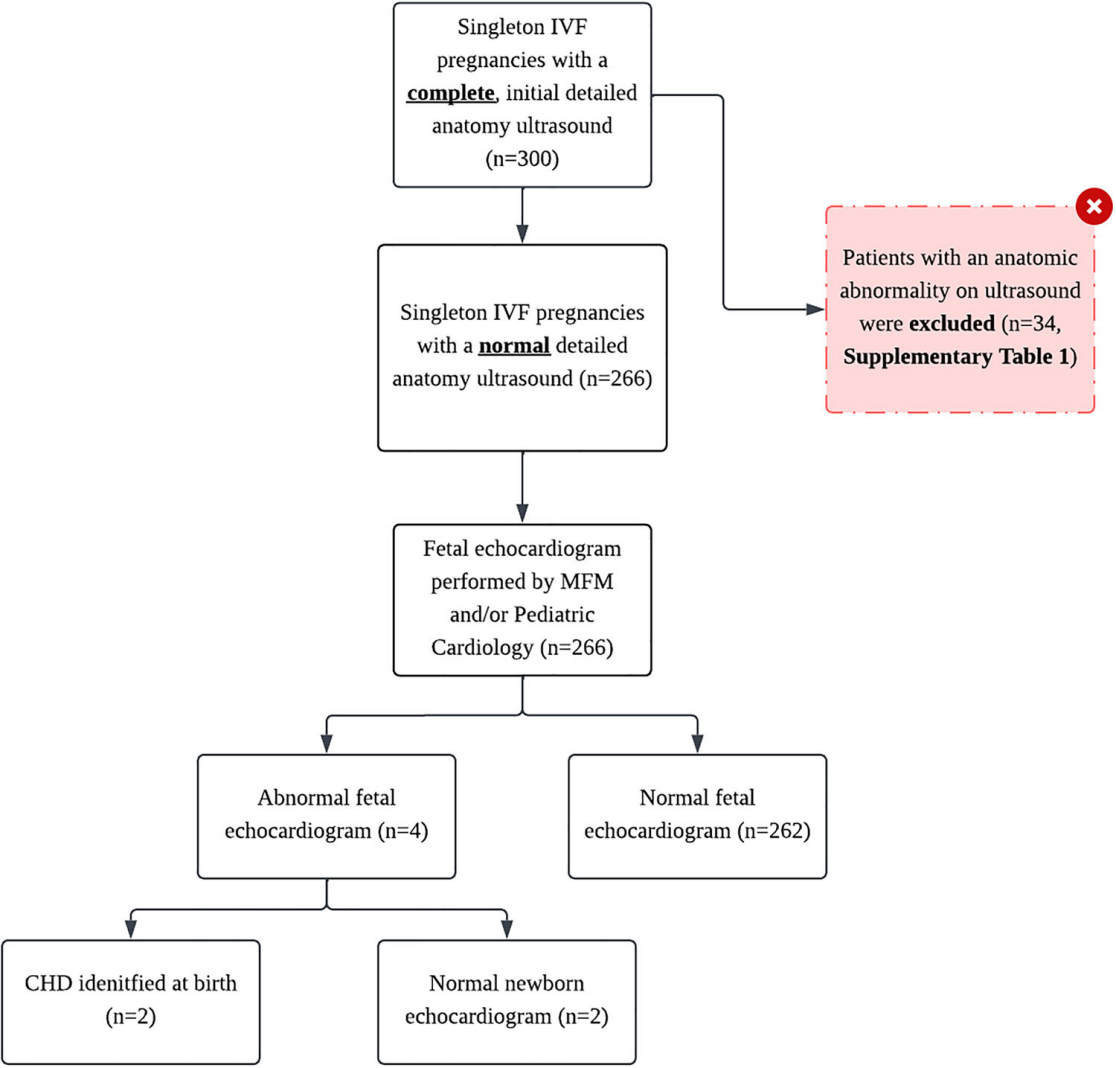
Study Design Flowchart. 300 patients met the study inclusion criteria and 34 patients were subsequently excluded for having an abnormal anatomy scan. 266 patients were included for study analysis. MFM, maternal‐fetal medicine; IVF, In‐Vitro fertilization; CHD, congenital heart disease.

The primary outcome was the rate of detection of fetal CHD using fetal echocardiogram among IVF pregnancies after a normal and complete detailed ultrasound examination. Normal and complete detailed ultrasound examination was defined as a second trimester detailed comprehensive obstetric ultrasound examination (CPT code 76811) which includes “satisfactory and normal” views of the heart (four chamber view including visualization of pulmonary veins, right and left ventricular outflow tracts, aortic and ductal arches, bicaval, three vessel and three vessel tracheal views) with no follow up anatomic evaluation recommended by MFM clinicians [14]. This comprehensive anatomic survey differs from the routine second/third trimester anatomic survey ultrasound (CPT code 76805) which is routinely indicated in all pregnancies without an indication for a more comprehensive fetal anatomic survey. The comprehensive ultrasound provides more detailed information on all fetal anatomic structures while also including all components of a routine second/third trimester anatomic survey [14]. Fetal echocardiography (CPT code 76825) was performed by MFM specialists and pediatric cardiologists in accordance with AIUM guidelines [2, 11, 15]. If a cardiac difference was suspected, it was confirmed in multiple sonographic images, with and without color flow Doppler, to minimize the risk of a color artifact being falsely identified as a cardiac difference.

A major cardiac anomaly or difference was defined as a diagnosis that modifies the birth plan (change in mode, venue, or need for specialized testing immediately after birth), or a diagnosis that is anticipated to require an intervention (surgery or interventional cardiac procedure such a cardiac catheterization) in the first year of life. A minor cardiac anomaly was defined as an isolated finding that did not alter delivery plans or require immediate medical intervention, but for which cardiology follow‐up with neonatal echocardiography after birth was still recommended. The presence of intermittent premature atrial contractions was not considered a cardiac anomaly or difference.

Demographic and obstetric characteristics were summarized for all patients. Timing and location of fetal echocardiogram and the presence or absence of cardiac anomalies or other fetal anomalies at anatomic ultrasound or fetal echocardiogram, were summarized. To investigate the association between findings of any anomalies on complete detailed ultrasound examination and the primary outcome, Chi‐square test was used. To investigate the association between continuous variables and anomalies identified on complete fetal anatomy screening ultrasound, Wilcoxon rank‐sum test was used; Chi‐square or Fisher's exact tests were used for categorical variables while Student's t‐test and Wilcoxon rank‐sum test were used for continuous variables. All reported p‐values are two‐sided and a significance level of 0.05 was used. Statistical analyses were performed using R (version 4.6.0, R Core Team).

## 3 Results

There were 300 IVF singleton pregnancies that met the inclusion and exclusion criteria, with their demographics summarized in Table 1. Of this group, 266 pregnancies had a complete and normal detailed ultrasound examination performed by MFM at the time of the initial ultrasound visit. 34 pregnancies were excluded due to the identification of a cardiac anomaly or arrhythmia (*n* = 8, 2.6% of the total cohort), or non‐cardiac structural differences, including soft markers of aneuploidy (*n* = 26, 8.6% of the total cohort) as seen in Table S1. Of pregnancies with a normal and complete detailed ultrasound, including normal cardiac structures, four (1.5%) had CHD confirmed on fetal echocardiogram (Table 2).

**TABLE 1:** Summary of patient demographics.

Variable	All patients (*N* = 300)
Age at delivery, mean (SD)^a^	36.0 (4.6)
Race/ethnicity, *n* (%)^b^	
Non‐Hispanic White	255 (86.4)
Non‐Hispanic Black	7 (2.4)
Hispanic	1 (0.3)
Asian	24 (8.1)
Native American, Indigenous or Pacific Islander	4 (1.4)
Middle Eastern	0 (0.0)
More than 1 race/ethnicity	4 (1.4)
Pre‐pregnancy BMI, mean (SD) ^c^	24.9 (4.5)
Parity, *n* (%)^d^	
Nulliparous	174 (58.4)
Multiparous	124 (41.6)
IVF type, *n* (%)	
IVF	224 (74.7)
IVF with ICSI	12 (4.0)
IVF with donor egg	40 (13.3)
IVF with donor sperm	8 (2.7)
IVF with donor sperm and donor egg	14 (4.7)
IVF with ICSI and donor egg or sperm	2 (0.7)

**Abbreviations**: BMI, body mass index; ICSI, intracytoplasmic sperm injection; Q, quartile; SD, standard deviation.

^a^ Age is missing for 5 individuals.

^b^ Race is missing for 5 individuals.

^c^ BMI is missing for 17 individuals.

^d^ Parity is missing for 2 individuals.

**TABLE 2:** Summary of anatomy ultrasound and fetal echocardiography details.

Variable	Complete and normal anatomy ultrasound (*N* = 266)
GA at anatomy scan, median (Q1, Q3)	18.9 (18.4, 19.6)
GA at fetal echocardiogram, median (Q1, Q3)	22.1 (21.4, 22.9)
Location of fetal echocardiogram, *n* (%)	
MFM	228 (85.7)
Pediatric cardiology	34 (12.8)
Both	4 (1.5)
Cardiac anomaly at fetal echocardiogram, *n* (%)	4 (1.5)

**Abbreviations**: GA, gestational age; MFM, maternal‐fetal medicine; Q, quartile.

The four cases of CHD identified on fetal echocardiogram after normal and complete fetal anatomic surveys were defined as minor cardiac anomalies. The specific anomalies identified were three ventricular septal defects (VSDs) and an isolated interrupted inferior vena cava with azygous continuation without evidence of left atrial isomerism (Table 3). No additional cardiac anomalies were identified in the neonatal period and two of the pre‐natal VSDs were not identified at the time of the first neonatal echocardiogram within the first two weeks of life.

**TABLE 3:** Summary of cardiac anomalies.

Cardiac anomalies detected on fetal echocardiogram after normal and complete detailed ultrasound examination (*n* = 4, 1.5%)
1. Small peri membranous VSD, resolved by birth
2. Small apical VSD, resolved by birth
3. Small muscular VSD, present at birth
4. Interrupted IVC with azygous continuation, present at birth

**Abbreviations**: IVC, inferior vena cava; VSD, ventricular septal defect.

## 4 Discussion

In this cohort of IVF pregnancies, the detection rate of CHD on fetal echocardiogram after normal and complete detailed ultrasound examination was 1.5%, with a total identified CHD rate of 4% (12 of 300 completed anatomy evaluations) when fetal cardiac differences identified on comprehensive anatomy ultrasound were included as well. The rate reported in this study is in line with previously reported rates of CHD detection in this population (1.3%–8.5%) [5, 16, 17]. The reported rate of CHD detection in published data is slightly higher in pregnancies achieved using IVF than in all pregnancies in the general population, where the range is reported from 0.3% to 1.2% [5]. All four of the patients with normal fetal survey and subsequent diagnosis of CHD by fetal echocardiogram in this study had minor cardiac anomalies that did not require altered delivery plans or immediate medical interventions. In recently published results by Donovan et al. (2025) from a cohort of similar size, there was no difference in specificity between detailed anatomy ultrasound and fetal echocardiogram [18]. Similarly, no major cardiac anomalies were missed on complete anatomic survey at our center, suggesting that standard anatomy screening may be an adequate tool to rule out most major CHD in locations where echocardiogram may not be easily accessible. Our results lend support to the recently updated recommendations from the ASE and AIUM that a dedicated fetal echocardiogram may be considered, rather than recommended, for all IVF pregnancies in settings with access to detailed obstetric ultrasound examinations [11].

While the rate of CHD is still considered to be higher in pregnancies achieved by IVF, it is important to acknowledge that none of the detected minor cardiac anomalies, in this cohort and in similar research studies, required immediate intervention at the time of hospital birth or altered delivery plan [5, 16, 17]. VSDs may regress spontaneously or require surgical management and it is prudent to identify these prenatally with fetal echocardiogram, however in this case no neonatal cardiac intervention was required and two VSDs in the cohort did appear to self‐resolve [19]. The patient with interrupted inferior vena cava with azygous continuation did not require surgical or medical intervention during early childhood and was not identified to have left atrial isomerism, which can have more significant implications for long term health. Isolated interrupted inferior vena cava with azygous continuation is typically asymptomatic, though may pose a risk to unrelated thoracic surgical procedures if not previously identified and considered pre‐operatively [20].

Strengths of this study include data collection from a single system with standardized fetal echocardiogram protocols with study interpretation by fetal cardiologist or maternal fetal medicine specialists. In addition, all fetal anatomic surveys and echocardiograms were performed within the same medical record system as that used for the overwhelming majority of birth admissions and neonatal care in the area. Length of hospital stay and birth complications were able to be evaluated, allowing us to examine immediate clinical significance of any identified cardiac anomalies or differences. And although a small portion of pediatric patients were lost to follow up, we were able to follow all newborns with abnormal fetal echocardiography through early childhood to better understand the clinical significance of their CHD.

This study also has some limitations. This cohort included 300 pregnancies with completed initial detailed anatomy ultrasounds, which may not be a large enough sample to detect rare major cardiac anomalies. In addition, the patient population in this cohort was 86.4% non‐Hispanic white individuals, and all patients had access to detailed anatomic surveys with MFM specialists, which may limit generalizability. The detection rate of fetal cardiac anomalies on echocardiogram after normal and complete detailed ultrasound examination among non‐IVF pregnancies in this health system was not evaluated as a part of the study. In addition, the incidence of CHD statewide is also not reported as a comparison, as these data are not currently publicly available. However, there is little reason to suspect that either the incidence of CHD locally or the rate of detection of CHD after normal fetal anatomy screening amongst non‐IVF pregnancies would be drastically different compared to other states and health systems which have previously published on this topic [5].

In summary, our results add to the growing body of evidence supporting the new guidelines from the ASE stating that fetal echocardiograms “may be considered” depending on the sensitivity and specificity of CHD detection by detailed ultrasound examination [11]. Fetal echocardiogram may not be necessary for every pregnancy conceived by IVF, as a complete anatomic survey may effectively screen for major cardiac anomalies. However, routine screening of IVF pregnancies with fetal echocardiogram may still yield clinically important information in settings without consistent access to detailed ultrasound examination (CPT code 76811) or with an incomplete initial fetal detailed anatomic survey. Even in cases where routine ultrasonography is highly reliable, individual patient characteristics such as maternal pre‐pregnancy BMI may inform the decision of whether to move forward with fetal echocardiogram, although this was not examined in our study. Therefore, individual institutions may need to evaluate the reliability of their fetal ultrasound screening methods. Institutions may consider establishing formal ultrasound education curricula for their physicians in training and pursuing AIUM accreditation when seeking to maximize the sensitivity and specificity of their ultrasound screening practices [21, 22].

## 5 Conclusion

Fetal echocardiograms remain a potential component of the evaluation of fetal development in pregnancies achieved by IVF. In this cohort, no major cardiac anomalies were missed with a complete detailed obstetric ultrasound examination that was normal. This data may be interpreted with caution, as clinically significant findings may be missed without detailed evaluation of fetal cardiac anatomy by fetal echocardiogram. Further meta‐analysis or larger studies are likely required prior to further changes in fetal echocardiography guidelines, in order to allow for detection and evaluation of rare fetal cardiac differences. Obstetric care professionals should consider individual patient characteristics when ordering and discussing sonographic protocols for IVF pregnancies. Practitioners may utilize shared‐decision making in the context of the sensitivity and specificity of detailed ultrasound examination in their clinical setting.

## Funding Statement

Research reported in this publication was supported by the National Institutes of Health (NIH) grant P30 CA77598 utilizing the Biostatistics Core shared resource of the Masonic Cancer Center, University of Minnesota and by the National Center for Advancing Translational Sciences of the NIH Award Number UM1TR004405. The content is solely the responsibility of the authors and does not necessarily represent the official views of the National Institutes of Health.

## Ethical Approval Statement

This study was determined to meet the criteria for exemption from Institutional Review Board (IRB) review by the University of Minnesota IRB in Minneapolis, MN, USA on 11/20/2022 (IRB #STUDY00016221).

## Consent

This study included data from patients who provided informed consent to the use of their medical data in research.

## Conflicts of Interest

The authors have no conflicts of interest to declare.

## Supporting information




**Supporting Information**: echo70655‐sup‐0001‐SuppMat.docx

## Data Availability

The data supporting the findings of this study are not openly available due to reasons of human participant privacy and ethical restrictions. De‐identified data may be available from the authors upon reasonable request.
