# Supplementary material for: Fetal Echocardiography After a Normal Anatomy Assessment: A Retrospective Cohort of In Vitro Fertilization Pregnancies
Source: Echocardiography. 2026 Oct 24;43(10):e70655. doi: 10.1111/echo.70655 (PMC13695326; doi:10.1111/echo.70655)
Supplement: Supplementary file 1 [file ECHO-43-e70655-s001.docx]

**Supplementary Table 1.** Summary of Anomalies Identified on Complete Detailed Ultrasound Examination (n=34)

**Cardiac anomalies (n=8, 2.6% of total patients)**

Apical VSD (3)

Rhabdomyoma (1)

Atrial septal aneurysm (1)

Enlarged pulmonary outflow tract (1)*

Persistent Fetal Arrythmia (2)

**Noncardiac anomalies (n=26, 8.6% of total patients)**

Urinary tract dilation (9)

Choroid plexus cysts (4)

Cystic hygroma/thickened nuchal fold (3)

Club foot (2)

Absent nasal bone (2)

Cleft lip and palate, unilateral (1)

Two vessel cord (1)

Persistent right umbilical vein (1)

Umbilical vein varix (1)

Pleural effusion (1)

Liver calcifications (1)

* Found to be normal on fetal echocardiogram; VSD: Ventricular Septal Defect.
